# Clinical relevance of HLA-DQ eplet mismatch and maintenance immunosuppression with risk of allosensitization after kidney transplant failure

**DOI:** 10.3389/fgene.2024.1383220

**Published:** 2024-04-04

**Authors:** Jenny Tran, Ibrahim Alrajhi, Doris Chang, Karen R. Sherwood, Paul Keown, Jagbir Gill, Matthew Kadatz, John Gill, James H. Lan

**Affiliations:** ^1^ Department of Pathology and Laboratory Medicine, Faculty of Medicine, University of British Columbia, Vancouver, BC, Canada; ^2^ King Faisal Specialist Hospital and Research Center, Riyadh, Saudi Arabia; ^3^ Vancouver Coastal Health Research Institute, Faculty of Medicine, University of British Columbia, Vancouver, BC, Canada; ^4^ Division of Nephrology, Department of Medicine, Faculty of Medicine, University of British Columbia, Vancouver, BC, Canada; ^5^ Providence Health Care Research Institute, Faculty of Medicine, University of British Columbia, Vancouver, BC, Canada

**Keywords:** kidney transplant, epitope, eplet mismatch, transplant failure, allosensitization, immunosuppression, cPRA

## Abstract

The optimal immunosuppression management in patients with a failed kidney transplant remains uncertain. This study analyzed the association of class II HLA eplet mismatches and maintenance immunosuppression with allosensitization after graft failure in a well characterized cohort of 21 patients who failed a first kidney transplant. A clinically meaningful increase in cPRA in this study was defined as the cPRA that resulted in 50% reduction in the compatible donor pool measured from the time of transplant failure until the time of repeat transplantation, death, or end of study. The median cPRA at the time of failure was 12.13% (interquartile ranges = 0.00%, 83.72%) which increased to 62.76% (IQR = 4.34%, 99.18%) during the median follow-up of 27 (IQR = 18, 39) months. High HLA-DQ eplet mismatches were significantly associated with an increased risk of developing a clinically meaningful increase in cPRA (*p* = 0.02) and *de novo* DQ donor-specific antibody against the failed allograft (*p* = 0.02). We did not observe these associations in patients with high HLA-DR eplet mismatches. Most of the patients (88%) with a clinically meaningful increase in cPRA had both a high DQ eplet mismatch and a reduction in their immunosuppression, suggesting the association is modified by immunosuppression. The findings suggest HLA-DQ eplet mismatch analysis may serve as a useful tool to guide future clinical studies and trials which assess the management of immunosuppression in transplant failure patients who are repeat transplant candidates.

## Introduction

Transplant failure is the fourth leading cause for initiating dialysis in the U.S (Gill, 2011; Johansen et al., 2021). Patients with previous allograft failure have increased mortality on the wait-list compared to those with no prior failed transplant (Gill et al., 2002; Kaplan and Meier-Kriesche, 2002; Knoll et al., 2005; Rao et al., 2007; Kabani et al., 2014; Clark et al., 2019; Lam et al., 2020). While receipt of a repeat transplant can markedly improve survival, only 15% of patients with a failed transplant will receive a second transplant (Ojo et al., 1998; Rao et al., 2006; Rao et al., 2007; Schold et al., 2020). One of the main factors limiting repeat transplantation is allosensitization, the development of antibodies to human leukocyte antigens (HLA) expressed by donor kidneys (Lucisano et al., 2019). Since avoidance of preformed donor-specific antibodies (DSA) is the standard of practice in most transplant centers, sensitized candidates have decreased access to transplantation in relation to the breadth of their anti-HLA antibodies. Preventing allosensitization is therefore of critical importance to increase the probability of repeat transplantation for the thousands of patients who lose their kidney transplants annually.

Maintaining patients on their immunosuppressive drugs after transplant failure may reduce the risk of HLA antibody development. Several single-center studies report a significant increase in the calculated panel reactive antibody (cPRA) of transplant candidates who reduced their immunosuppression after allograft failure (Augustine et al., 2012; Casey et al., 2014; Nimmo et al., 2018; Lucisano et al., 2019); however, this finding has not been consistently reproduced in other studies (Martin et al., 2021; Knoll et al., 2022; Elgenidy et al., 2023). Potential reasons for this discrepancy include heterogeneity in the study cohorts, lack of granular immunosuppression data capture, differences in the methodology of HLA antibody detection and the thresholds for defining the outcome of allosensitization, and variable consideration for HLA mismatches. Currently, national transplant society and expert recommendations regarding immunosuppression management are not evidence based (Davis and Mohan, 2022), resulting in a wide variation of clinical practice (Fiorentino et al., 2020; Alhamad et al., 2021). An individualized approach is therefore urgently needed to risk stratify patients with transplant failure, weighing the potential benefits of maintaining immunosuppression against the risk of infections, malignancies, and cardiovascular complications (Pham et al., 2015).

Several studies have shown that the incremental number of mismatches at HLA loci is associated with allosensitization after transplant failure (Augustine et al., 2012; Del Bello et al., 2012). Furthermore, precise assessment of differences in the critical amino acids within the epitope of the HLA protein that are considered essential for antibody binding (eplets) has been shown to outperform traditional antigen matching methods in predicting outcomes after transplantation (Duquesnoy, 2006; Wiebe et al., 2017; Senev et al., 2020). Based on a recent observation that mismatched HLA-DQ antigens were the most likely to be listed as unacceptable in patients who re-entered the wait-list after transplant failure (Isaacson et al., 2022), we hypothesized that class II HLA eplet mismatches, particularly at the DQ locus, could serve as a practical risk stratification tool to guide the immunosuppression management of patients after graft failure. The primary objective of this study was thus to evaluate the impact of class II eplet mismatches on the risk of allosensitization and the likelihood of re-transplantation in a well characterized cohort of transplant failure patients with granular immunosuppressive data.

## Materials and methods

### Study cohort

The study cohort included 21 kidney transplant recipients in Vancouver, Canada who were wait-listed for a second kidney transplant following allograft failure and had available donor and recipient DNA for high resolution HLA genotyping at the HLA-DRB1, DRB3, DRB4, DRB5, DQA1, and DQB1 genes. Dates of the primary transplant ranged from January 1998–September 2013. Transplant failure was defined as resumption of chronic dialysis. None of the study patients received pre-emptive transplantation following a failed first transplant. At the end of study follow-up (August 2022), 15 patients received a second transplant, three patients died on the wait-list, and another three patients remained on the wait-list. All patients had stored sera prior to or within 1 month of transplant failure for determination of baseline cPRA. The last follow-up serum sample was collected prior to the time of repeat transplantation or end of study follow-up. Clinical data including the use of maintenance immunosuppression and calcineurin inhibitor drug levels were retrieved from electronic health records. This study was approved by UBC Research Ethics Board H22-02521.

### HLA typing and eplet mismatches

High-resolution HLA genotyping was performed using Next-Generation Sequencing (Holotype HLA, Omixon, Budapest) and the MiSeq (Illumina, California, USA). HLA eplet mismatches were determined between each transplant pair using R v4.1.3 with the eplet database from HLAMatchmaker v2 (Duquesnoy, 2006). Eplet mismatches were determined for HLA class I comprising HLA-A, B, and C eplets, HLA-DR comprising HLA-DRB1, DRB3, DRB4, DRB5 eplets, HLA-DQ comprising HLA-DQA1 and DQB1 eplets, and HLA-DP comprising HLA-DPA1 and DPB1 eplets. Thresholds for eplet mismatches were determined using previously published data (Wiebe et al., 2017). A low DR mismatch was defined as 0–11 eplet mismatches and low DQ mismatch was 0–11 eplet mismatches. A high DR mismatch was 12 or more eplet mismatches and a high DQ mismatch was 12 or more eplet mismatches.

### HLA antibody testing

HLA antibody testing included an initial screen using LABScreen PRA Class I/II (OneLambda, Canoga Park, California, USA), and upon a positive result, the precise identification of antibodies was performed using LABScreen Single Antigen HLA Class I/II (OneLambda, Canoga Park, California, United States). Consistent with Canadian HLA Laboratory policy guidelines, a mean fluorescent intensity (MFI) threshold of >1,000 was used to define unacceptable antigens (Canadian Blood Services, 2018). Cumulative antibodies detected at the HLA-A, B, Cw, DRB1, DRB3, DRB4, DRB5, DQA1, DQB1, DPA1, and DPB1 loci were used to compute patients’ cPRA using the Canadian Blood Services (CBS) cPRA calculator (www.pra-calculator.ca) (Canadian Blood Services, 2019). During wait-listing, patients were tested for antibodies at a frequency based on a standardized local protocol: 6 months for highly sensitized patients (HSP) defined as cPRA ≥94.50%; annually for non-HSP patients; and 3 months for patients enrolled in the kidney paired donation program. The change in cPRA after transplant failure was calculated for all patients by subtracting the patient’s baseline cPRA at the time of transplant failure from the last recorded cPRA measured at the time of repeat transplantation or end of study.

**FIGURE 1:**
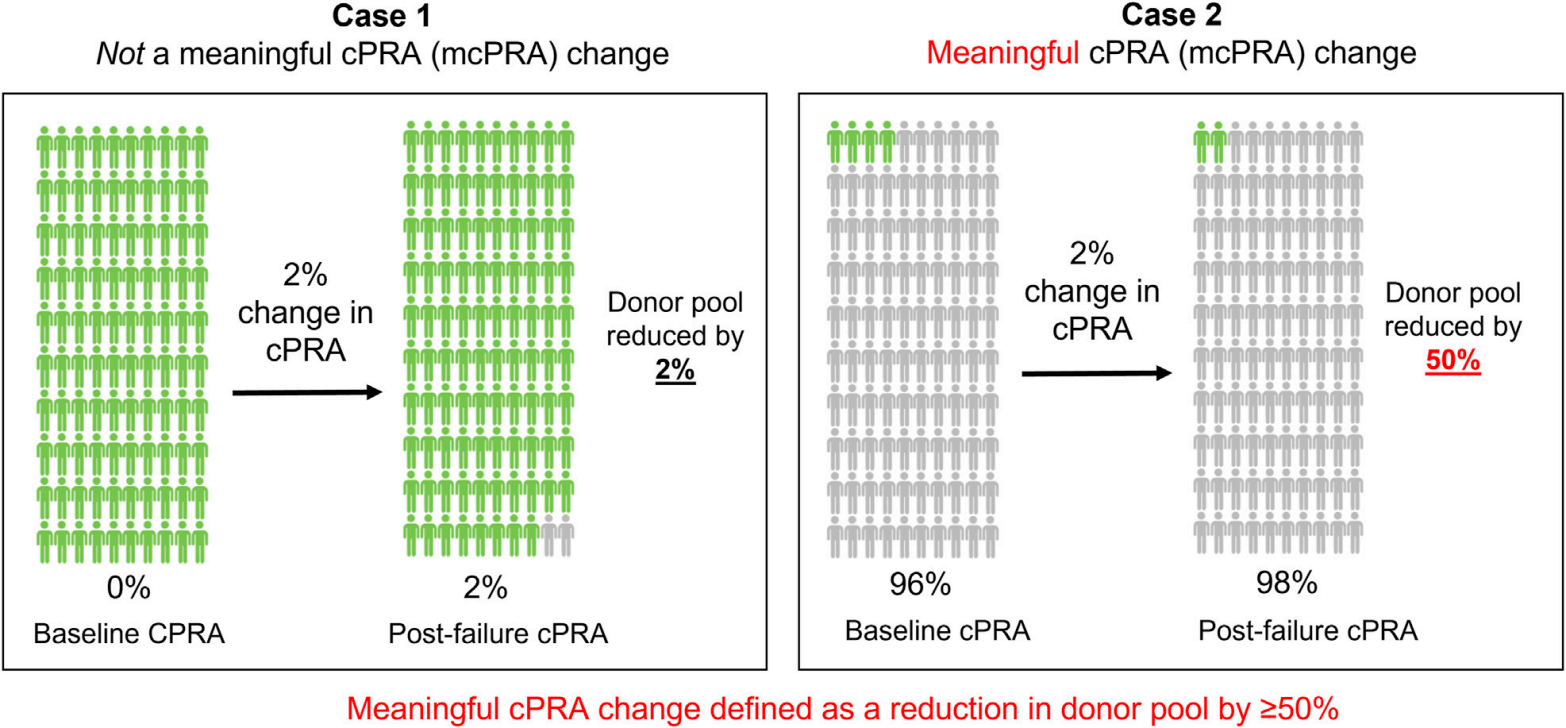
A meaningful cPRA (mcPRA) increase. The magnitude of change in cPRA after transplant failure can have a differential impact on access to transplantation depending on the cPRA at the point of failure (baseline). In Case 1, a 2% increase in cPRA in a patient with a baseline cPRA of 0% results in a compatible donor pool reduction by 2%. In contrast, Case 2 represents the same 2% change in cPRA in a different patient with a baseline cPRA of 96%, but this results in a compatible donor pool reduction of 50%. To account for this effect, we defined a meaningful cPRA (mcPRA) change in this study as a 50% or greater reduction in donor pool availability after transplant failure. Based on this definition, the cPRA threshold for reaching a meaningful change is 50% in Case 1% and 98% in Case 2.

The magnitude of change in cPRA can have a differential impact on access to transplantation depending on the baseline cPRA at the time of transplant failure (Figure 1). To account for this effect, we defined the outcome of a meaningful cPRA (mcPRA) increase in this study as the increase in cPRA that resulted in a 50% reduction in the size of the compatible donor pool between the baseline measure (prior to or within 1 month of the date of transplant failure) and the last follow-up sample. This metric has recently been utilized in other studies to quantify secular changes in cPRA (Vincenti et al., 2023).

### Statistical methods

Numerical variables were described as medians with interquartile ranges (IQR) and categorical variables as counts and proportions. Fisher’s exact test was used to compare patients with low and high eplet mismatch on *de novo* donor-specific antibody (dnDSA) and mcPRA as well as antigen and allelic mismatch on mcPRA. Two-sample *t*-test was used to compare the change in cPRA during follow-up in patients with low and high eplet mismatches as well as the effect of eplet mismatch sum on a mcPRA change.

## Results

The study cohort was multi-ethnic, comprising 42.86% White, 28.57% Asian Indian, and 28.57% Southeast/East Asian. The cohort was 38% female and the median age at transplant was 33 (IQR = 28, 54). Allograft failure occurred at a median time of 88 (IQR = 50, 123) months post-transplant. The median follow-up for cPRA testing after transplant failure was 27 (IQR = 18, 39) months.

The median cPRA at the time of failure (baseline) was 12.13% (IQR = 0.00%, 83.72%). Eight patients (38%) had a baseline cPRA = 0.00%, 11 patients (52%) with a cPRA between >0.00% and <94.50%, and two patients (10%) were in the Canadian highly sensitized registry with a baseline cPRA ≥94.50%. During follow-up, the cPRA increased to a median of 62.76% (IQR = 4.34%, 99.18%) post-failure.

**FIGURE 2:**
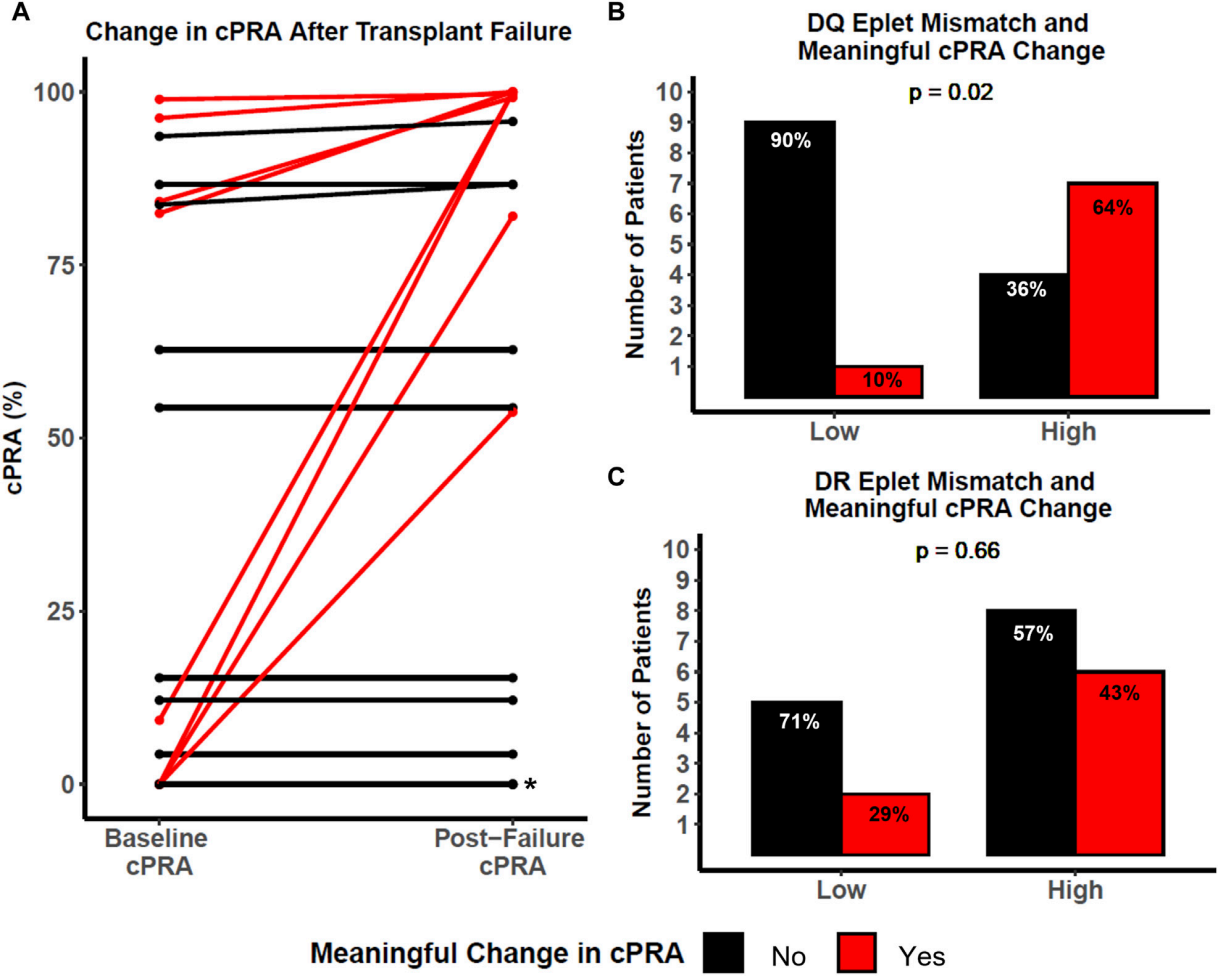
The change in cPRA after transplant failure. **(A)** All patients and their cPRA at the point of failure (baseline) plotted on the left and their corresponding cPRA after failure on the right. Connections represent the same patient and their change in cPRA. Patients with a meaningful cPRA (mcPRA) change are in red, and those that are not in black. *Represents five patients whose baseline cPRA was 0.00% and remained 0.00% after failure. **(B)** Patients stratified by low or high DQ eplet mismatch and whether they experienced a mcPRA change after transplant failure in red or not in black. **(C)** Patients stratified by low or high DR eplet mismatch and whether they experienced a mcPRA change after transplant failure in red or not in black. *p* values calculated using Fisher’s exact test.

Among the 21 study patients, ten (48%) had an increase in cPRA, where eight (38%) recipients had a mcPRA change after transplant failure (Figure 2A). Five out of 19 (26%) patients who were previously not highly sensitized became highly sensitized with cPRA ≥94.5% after failure. Eleven (52%) patients did not have a change in their cPRA: five (24%) patients remained at a cPRA of 0.00% pre- and post-transplant failure. The median time of follow-up for calculating cPRA change was longer for patients that had mcPRA change *versus* those that did not (55 (IQR = 22, 80) vs 24 (IQR = 18, 30) months). However, the time to reach a mcPRA change was a median of only 19 (IQR = 10, 31) months.

In this cohort, patients with high DQ eplet mismatches had a median cPRA change of 15.02% (IQR = 1.08%, 67.90%) and patients with low DQ eplet mismatches had a median cPRA change of 0.00% (IQR = 0.00%, 0.00%). The average cPRA change was significantly higher in patients with high DQ eplet mismatches than those with low DQ mismatches (*p* = 0.02). Seven out of eleven (64%) patients with high DQ eplet mismatches developed a mcPRA change post-failure compared with only 1/10 (10%) patients with low DQ eplet mismatches (*p* = 0.02) (Figure 2B). In contrast, high DR eplet mismatches did not associate with an overall increase in cPRA (*p* = 0.70) or mcPRA change (*p* = 0.66) (Figure 2C). When analyzed by the sum of eplet mismatches, increasing number of eplet mismatches was similarly associated with a mcPRA change for only DQ (*p* = 0.01), but not DR (*p* = 0.54), class I (*p* = 0.20), or DP (*p* = 0.86) (Supplementary Figure S1). Furthermore, neither antigen nor allelic-level mismatches significantly associated with a meaningful cPRA change across all 11 HLA loci tested (Supplementary Figures S2, S3).

There were two patients with allelic *match* (i.e., zero eplet mismatch) at DQA1 and DQB1 against their donor and one patient with allelic *match* at DRB1, DRB3, DRB4, and DRB5. Exclusion of these patients did not change the study finding that patients with high DQ eplet mismatch were more likely to have a mcPRA change (*p* = 0.01), and this association remained non-significant for patients with high DR eplet mismatch (*p* = 0.35) (Supplementary Figure S4). Similarly, the magnitude of change in cPRA remained statistically significant for DQ (*p* = 0.04) but not for DR (*p* = 0.82).

**FIGURE 3:**
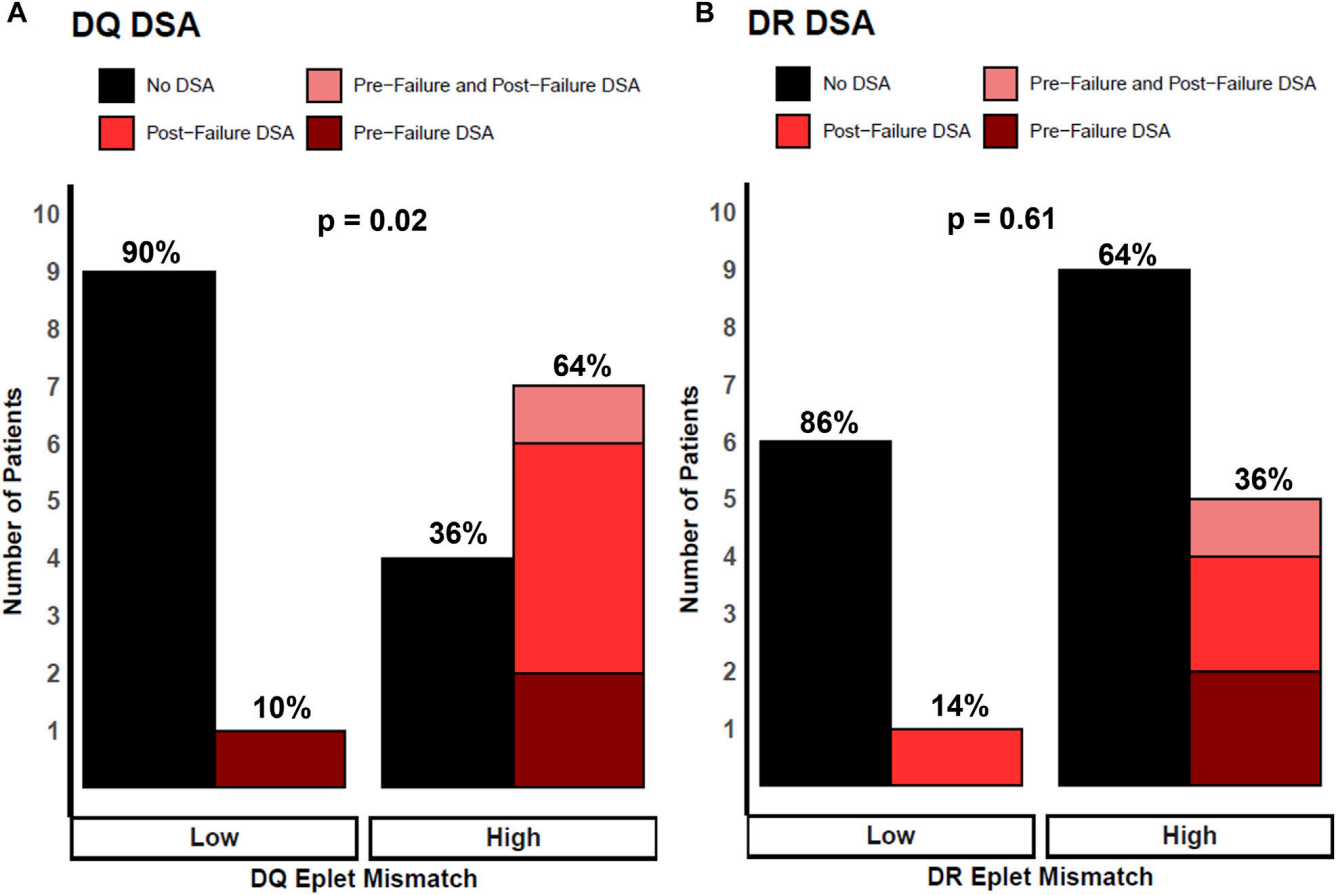
Patients stratified by low and high eplet mismatch and locus-specific dnDSA development after transplant. Height of bars represents number of patients. Black bars represent no DSA. Colored red bars represent DSA development post-transplant, where light red are patients with DSA pre-failure and post-failure, bright red as patients with DSA post-failure, and dark red as patients with DSA pre-failure. Percentages represent proportion of patients within each eplet mismatch risk group. **(A)** Patients stratified by DQ eplet mismatch and DQ dnDSA development. **(B)** Patients stratified by DR eplet mismatch and DR dnDSA development. *p* values calculated using Fisher’s exact test.

Figure 3 shows the association of class II eplet mismatches and the development of dnDSA post-transplant (pre-failure and post-failure). For DQ, eight (38%) patients developed dnDSA after transplant, with three (37.5%) detected *pre-failure*, four (50%) occurring *post-failure*, and one (12.5%) patient developed DQ dnDSA both *pre- and post-transplant failure* (Figure 3A). For DR, six (29%) patients had dnDSA after transplant: two (33%) developed DR dnDSA *pre-failure*, three (50%) *post-failure*, and one (17%) patient developed DR dnDSA *both pre- and post-transplant failure* (Figure 3B). Overall, patients with high DQ eplet mismatches were statistically significantly more likely to develop post-transplant DQ dnDSA compared with patients with low DQ mismatches (64% vs 10%, *p* = 0.02). All recipients (5/5) who developed DQ dnDSA *post-failure* had high DQ eplet mismatch whereas 0/10 patients with low DQ eplet mismatch developed DQ dnDSA post-failure. Patients with high DR eplet mismatches trended toward development of post-transplant DR dnDSA compared to those with low mismatches but this did not reach statistical significance (36% vs 14%, *p* = 0.61).

**FIGURE 4:**
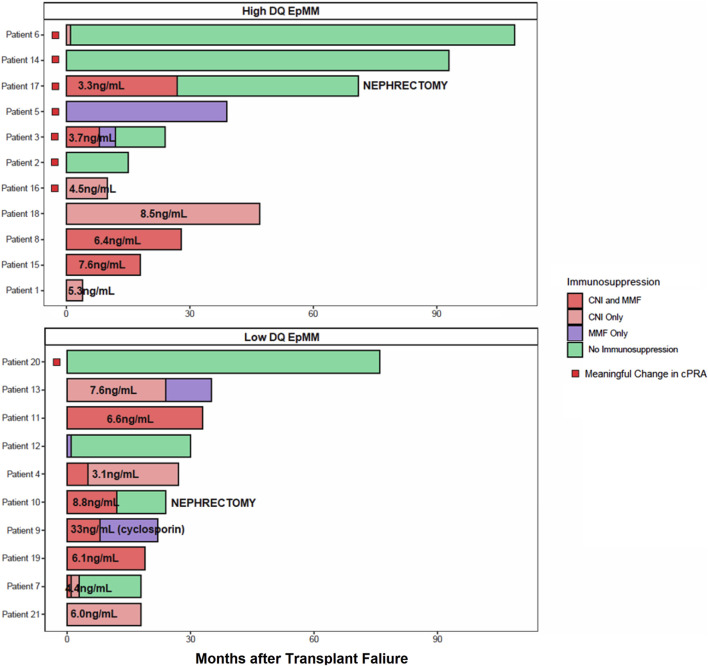
Immunosuppression medication use and DQ eplet mismatch on mcPRA change. Patients were stratified by high (top) *versus* low (bottom) DQ eplet mismatch. Each row represents a patient and the timeline of their immunosuppression use after transplant failure. Last month of follow-up was represented by their last recorded cPRA post-failure. Immunosuppression type is color-coded, where combined calcineurin-inhibitor (CNI) with mycophenolate mofetil (MMF) use are in dark red, CNI monotherapy in light red, MMF monotherapy in purple, and complete withdrawal of immunosuppression in green. All CNI was tacrolimus unless otherwise indicated as cyclosporin. Average CNI trough levels indicated as ng/mL on relevant bar graphs. Patients with a mcPRA change indicated by a red box. Patients who received a transplant nephrectomy are indicated by text.

Figure 4 shows the relationship of immunosuppressive medication use and DQ eplet mismatch with the development of a mcPRA change post-failure. Each patient’s maintenance immunosuppressive use after transplant failure was categorized as one of the following: mycophenolate mofetil (MMF) monotherapy; calcineurin-inhibitor (CNI) monotherapy with average CNI trough levels (all patients were on tacrolimus except for Patient nine who was on cyclosporin); combined CNI and MMF use; and complete withdrawal of immunosuppression. Most of the patients (7/8, 88%) with a meaningful cPRA change had both a high DQ eplet mismatch and a reduction in their immunosuppression. For example, Patient six who developed a mcPRA change had a high DQ eplet mismatch and underwent a complete immunosuppression withdrawal within 1 month post-failure. The four patients (e.g., 1, 8, 15, and 18) that had high DQ eplet mismatch but did not have a mcPRA change were all maintained on tacrolimus and had average trough levels ≥ 5 ng/mL. In contrast, the immunosuppressive management in recipients with a low DQ eplet mismatch was highly variable. Despite this, there was no mcPRA change with both high and low immunosuppression intensity with the exception of Patient 20 that had a low DQ eplet mismatch but experienced a mcPRA change. This patient had complete withdrawal of immunosuppression and their dnDSA was targeted against DP, leading to a cPRA changed from 98.92% to 99.67% post-failure. Two patients (e.g., 10 and 17) underwent allograft nephrectomy post-failure followed by complete immunosuppression withdrawal. Interestingly, one patient had a mcPRA change and one did not. Patient 17 with the mcPRA change (cPRA 9.24%–99.96%) had a high DQ eplet mismatch while Patient 10 with a low DQ eplet mismatch had no change to cPRA (remained at 86.61%) post-nephrectomy.

## Discussion

This study explored an innovative method of using HLA-DQ eplet mismatch to inform the risk of allosensitization in patients with a first kidney transplant failure. The principal finding of this study is that high HLA-DQ eplet mismatch was significantly associated with development of DQ dnDSA and a meaningful change in cPRA after transplant failure, and that this effect was modified by the intensity of maintenance immunosuppression post-failure.

In recent years, numerous studies have found an association between class II HLA eplet mismatches and adverse post-transplant outcomes, including development of dnDSA, allograft rejection, and inferior transplant survival (Wiebe et al., 2015; Wiebe et al., 2017; Wiebe et al., 2018; Wiebe et al., 2019; Senev et al., 2020). However, there are limited studies evaluating eplet mismatches and allosensitization *after* a failed allograft. In a study by Kosmoliaptsis et al., different mismatching methods including eplet mismatches were used to predict the risk of allosensitization following failure of a kidney transplant (Kosmoliaptsis et al., 2016). Despite the pivotal observation that amino acid sequence, eplet, and physicochemical disparities were more predictive of allosensitization post-failure across HLA-A, B, C, DR, DQ loci compared with HLA antigen mismatch, the study findings could not be easily translated into clinical practice due to the lack of an identified mismatch threshold to define low *versus* high risk. In addition, the study did not address which of the HLA loci when mismatched had the most contribution to a cPRA rise post-failure, which is an important consideration when designing a biomarker-driven pathway for immunosuppressive management after transplant failure. In the present study, HLA-DQ eplet mismatches were significantly associated with the development of DQ *de novo* DSA which occurred both pre-failure and post-failure. Other studies have shown that dnDSA at HLA-DQ are the most common post-transplant (Devos et al., 2012; Willicombe et al., 2012; Lee et al., 2016). A recent registry analysis involving 4,867 patients with a failed first kidney transplant also found that mismatched HLA-DQ antigens were the most clinically relevant in triggering allosensitization after graft failure (Isaacson et al., 2022). Together, these data suggest that risk stratification based on HLA-DQ eplet mismatch alone may adequately capture most patients at risk of developing clinically significant allosensitization without the restrictive strategy of considering all HLA loci.

Most of the studies which evaluated allosensitization after transplant failure used PRA or cPRA change as the primary study outcome (Augustine et al., 2012; Casey et al., 2014; Kosmoliaptsis et al., 2016; Martin et al., 2021; Knoll et al., 2022). However, these studies used a heterogenous definition that ranged from a class I or class II PRA >80% based on the less sensitive FlowPRA beads (Augustine et al., 2012) to cPRA ≥85% defined using the Luminex single antigen bead test (Kosmoliaptsis et al., 2016) which complicates interpretation of findings. Apart from the confounding issue of methodological differences in antibody testing technology, the non-linear relationship of cPRA with the compatible donor pool size means that the same absolute change in cPRA value has a different interpretation depending on the patient’s baseline cPRA (Lan, 2024). Applying a fixed cPRA threshold to define clinically significant sensitization will also fail to capture the profound clinical impact of a minor increase in cPRA in patients that are already highly sensitized at baseline (i.e., moving from 99.90% to 99.99%). In this study, we defined a meaningful cPRA change as one that would reduce the compatible donor pool by 50%, a criterion that was recently used as one of the key outcome measures in an international desensitization trial (Vincenti et al., 2023). Using this method, we were able to interpret the cPRA change of each patient on the same scale independent of their baseline cPRA. We found that all but one patient that had a meaningful change in cPRA had a high DQ eplet mismatch with their donor. Interestingly, this patient with low DQ eplet mismatch had complete withdrawal of immunosuppression immediately after graft failure and developed antibodies targeting the DEAV epitope which is commonly expressed on a broad array of HLA-DP antigens. This case highlights the importance of considering additional factors such as the timing and pace of immunosuppression withdrawal which can further modify the risk of allosensitization. Furthermore, although HLA-DQ appears to be the most at risk-locus in precipitating allosensitization, consideration of immunogenic targets from other loci might be required to further refine the precision of immunosuppressive management for each patient.

Despite the small sample size of this study, we were able to leverage the granularity of immunosuppression data to evaluate the impact of drug regimen and dose on risk of allosensitization. In recipients with high DQ eplet mismatch that experienced a mcPRA change, all had a reduction in their immunosuppression therapy either in the form of complete immunosuppression withdrawal, CNI cessation, or a reduction in the average tacrolimus trough level <5 ng/mL. In contrast, recipients with high DQ eplet mismatches but did not have a mcPRA change all maintained a tacrolimus trough level ≥5 ng/mL. These results were consistent with the study by Wiebe et al. that found that a tacrolimus trough level <5 ng/mL was the threshold in which the risk of dnDSA was increased in patients post-transplant (Wiebe et al., 2017). These results also corroborate with another study by the same group that found an association of class II eplet mismatch with suboptimal immunosuppression on rejection and graft loss (Wiebe et al., 2015). Notably, most recipients with low DQ eplet mismatch did not experience a mcPRA change regardless of their immunosuppression. Although these observations need to be confirmed in larger studies, these findings suggest that the risk of allosensitization associated with high DQ eplet mismatch could be attenuated by the intensity of maintenance immunosuppression and thus should be viewed as a modifiable risk factor.

There are several limitations that the reader should consider when interpreting the findings of this study. One study limitation relates to the variability of time elapsed between baseline and the last cPRA test performed. Although the median time between cPRA testing was longer in patients that developed a meaningful change in cPRA (55 months) compared to patients that did not have significant allosensitization (24 months), the time it took to reach the threshold of a meaningful cPRA change was only 19 months, and thus likely did not confound the interpretation of study findings. The lack of access to transfusion data did not allow us to evaluate the potential contribution of transfusion to allosensitization. Despite this limitation, we note that most of the patients (7/8) with a meaningful change in cPRA had demonstrable DSA that formed post-failure. In addition, none of the patients with low DQ eplet mismatch developed significant allosensitization due to third-party anti-HLA antibodies, indicating that the confounding impact of transfusion was likely minor in this study. Furthermore, the current Canadian Blood Services cPRA calculator does not include allelic antibodies in its calculation and thus the contribution of allelic antibodies to cPRA were not accounted for in this analysis. Despite this limitation, there were only two patients in this study with allelic antibodies which developed on follow-up post-failure and both patients reached cPRA = 100% after graft failure. Thus, consideration of these allelic antibodies would not have contributed to a further increase their cPRA and change the results of the analysis, but the potential impact of allelic antibodies to cPRA change should be considered for future studies.

It is also important to note that the eplet mismatch sum and the thresholds used for defining low or high DR/DQ eplet mismatch are based on statistical association with post-transplant outcomes as demonstrated in previous studies (Wiebe et al., 2017) and do not imply mechanistic underpinning such as eplet immunogenicity, physiochemical properties, and T cell epitopes which are likely involved in the generation of the allo-immune response in the transplant failure patient. Indeed, other mismatch algorithms may be used to supplement HLAMatchmaker-defined B cell eplet mismatches to ascertain patient-donor compatibility, including PIRCHE (Otten et al., 2013) and EMMA (Kramer et al., 2020), that could be explored in future studies with a larger cohort. The relatively small sample size of our study restricts the consideration of other factors that may affect cPRA, including recipient age, ethnicity, and duration of exposure to dialysis which can affect immune competency, as well as patient homozygosity, and frequency of antigens in the donor population. The study results should thus be taken with caution until they are validated in large studies which have the appropriate sample size to evaluate the other variables not included here. Notwithstanding these considerations, the granularity of the antibody and immunosuppression data coupled with the use of a novel approach in defining a clinically relevant allosensitization outcome demonstrate encouraging results to explore the potential use of HLA-DQ eplet mismatches in preventing allosensitization after a failed transplant.

In conclusion, HLA-DQ eplet mismatch is associated with allosensitization in patients with a failed renal transplant and this effect was modified by the intensity of maintenance immunosuppression. Eplet mismatch analysis may serve as a useful tool to guide future clinical studies and trials which assess the management of immunosuppression in transplant failure patients who contemplate repeat transplantation.

## Data Availability

The datasets presented in this article are not readily available because they are confidential health services data from British Columbia. Requests to access the datasets should be directed to JL on behalf of the British Columbia Transplant Organization. Requests to access the datasets should be directed to JL, james.lan@vch.ca.

## Supplementary material

SUPPLEMENTARY FIGURE S1 The effect of eplet mismatch sum on a meaningful cPRA (mcPRA) change. Patients experiencing a mcPRA change after transplant failure indicated in red or not in black. **(A)** DQ eplet mismatch. **(B)** DR eplet mismatch. **(C)** Class I (A, B, C) eplet mismatch. **(D)** DP eplet mismatch. *p* values calculated using Two-Sample T Test.

SUPPLEMENTARY FIGURE S2 The change in cPRA after transplant failure by antigen mismatch. Patients stratified by 0 or more than 0 (i.e., 1 or 2) antigen mismatch and whether they experienced a mcPRA change after transplant failure in red or not in black. **(A)** HLA-A antigen mismatch. **(B)** HLA-B antigen mismatch. **(C)** HLA-C antigen mismatch. **(D)** HLA-DQA1 antigen mismatch. **(E)** HLA-DQB1 antigen mismatch. **(F)** HLA-DRB1 antigen mismatch. **(G)** HLA-DRB3 antigen mismatch. **(H)** HLA-DRB4 antigen mismatch. **(I)** HLA-DRB5 antigen mismatch. **(J)** HLA-DPA1 antigen mismatch. **(K)** HLA-DPB1 antigen mismatch. *p* values calculated using Fisher’s exact test.

SUPPLEMENTARY FIGURE S3 The change in cPRA after transplant failure by allelic mismatch. Patients stratified by 0 or more than 0 (i.e., 1 or 2) allelic mismatch and whether they experienced a mcPRA change after transplant failure in red or not in black. **(A)** HLA-A allelic mismatch. **(B)** HLA-B allelic mismatch. **(C)** HLA-C allelic mismatch. **(D)** HLA-DQA1 allelic mismatch. **(E)** HLA-DQB1 allelic mismatch. **(F)** HLA-DRB1 allelic mismatch. **(G)** HLA-DRB3 allelic mismatch. **(H)** HLA-DRB4 allelic mismatch. **(I)** HLA-DRB5 allelic mismatch. **(J)** HLA-DPA1 allelic mismatch. **(K)** HLA-DPB1 allelic mismatch. *p* values calculated using Fisher’s exact test.

SUPPLEMENTARY FIGURE S4 The change in cPRA after transplant failure in patients >0 DQA1+DQB1 **(A)** or >0 DRB1+DRB3+DRB4+DRB5 **(B)** allelic mismatch. **(A)** Patients stratified by low or high DQ eplet mismatch and whether they experienced a mcPRA change after transplant failure in red or not in black. **(B)** Patients stratified by low or high DR eplet mismatch and whether they experienced a mcPRA change after transplant failure in red or not in black. *p* values calculated using Fisher’s exact test.

## References

[B1] Alhamad T. Lubetzky M. Lentine K. L. Edusei E. Parsons R. Pavlakis M. (2021). Kidney recipients with allograft failure, transition of kidney care (KRAFT): a survey of contemporary practices of transplant providers. Am. J. Transplant. 21 (9), 3034–3042. 10.1111/ajt.16523 33559315

[B2] Augustine J. J. Woodside K. J. Padiyar A. Sanchez E. Q. Hricik D. E. Schulak J. A. (2012). Independent of nephrectomy, weaning immunosuppression leads to late sensitization after kidney transplant failure. Transplantation 94 (7), 738–743. 10.1097/TP.0b013e3182612921 22955228

[B3] Canadian Blood Services (2018). Donation and transplantation guidelines and protocols.

[B4] Canadian Blood Services (2019). Canadian cPRA Calculator. Available from: https://ctr2.transplantregistry.ca/otd-cpra-client/ctr2.jsp.

[B5] Casey M. J. Wen X. Kayler L. K. Aiyer R. Scornik J. C. Meier-Kriesche H. U. (2014). Prolonged immunosuppression preserves nonsensitization status after kidney transplant failure. Transplantation 98 (3), 306–311. 10.1097/TP.0000000000000057 24717218 PMC4122584

[B6] Clark S. Kadatz M. Gill J. Gill J. S. (2019). Access to kidney transplantation after a failed first kidney transplant and associations with patient and allograft survival: an analysis of national data to inform allocation policy. Clin. J. Am. Soc. Nephrol. 14 (8), 1228–1237. 10.2215/CJN.01530219 31337621 PMC6682813

[B7] Davis S. Mohan S. (2022). Managing patients with failing kidney allograft many questions remain. Clin. J. Am. Soc. Nephrol. 17 (3), 444–451. 10.2215/CJN.14620920 33692118 PMC8975040

[B8] Del Bello A. Congy-Jolivet N. Sallusto F. Guilbeau-Frugier C. Cardeau-Desangles I. Fort M. (2012). Donor-specific antibodies after ceasing immunosuppressive therapy, with or without an allograft nephrectomy. Clin. J. Am. Soc. Nephrol. 7 (8), 1310–1319. 10.2215/CJN.00260112 22626959 PMC3408115

[B9] Devos J. M. Gaber A. O. Knight R. J. Land G. A. Suki W. N. Gaber L. W. (2012). Donor-specific HLA-DQ antibodies may contribute to poor graft outcome after renal transplantation. Kidney Int. 82 (5), 598–604. 10.1038/ki.2012.190 22622504

[B10] Duquesnoy R. J. (2006). A structurally based approach to determine HLA compatibility at the humoral immune level. Hum. Immunol. 67 (11), 847–862. 10.1016/j.humimm.2006.08.001 17145365 PMC2169290

[B11] Elgenidy A. Shemies R. S. Atef M. Awad A. K. El-Leithy H. H. Helmy M. (2023). Revisiting maintenance immunosuppression in patients with renal transplant failure: early weaning of immunosuppression versus prolonged maintenance—systematic review and meta-analysis. J. Nephrol. 36 (2), 537–550. 10.1007/s40620-022-01458-y 36109426

[B12] Fiorentino M. Gallo P. Giliberti M. Colucci V. Schena A. Stallone G. (2020). Management of patients with a failed kidney transplant: what should we do? Clin. Kidney J. 14, 98–106. 10.1093/ckj/sfaa094 33564409 PMC7857798

[B13] Gill J. S. (2011). Managing patients with a failed kidney transplant: how can we do better? Curr. Opin. Nephrol. Hypertens. 20 (6), 616–621. 10.1097/MNH.0b013e32834bd792 21946163

[B14] Gill J. S. Abichandani R. Khan S. Kausz A. T. Pereira B. J. G. (2002). Opportunities to improve the care of patients with kidney transplant failure. Kidney Int. 61 (6), 2193–2200. 10.1046/j.1523-1755.2002.00373.x 12028460

[B15] Isaacson D. Schold J. D. Gmeiner M. W. Copley H. C. Kosmoliaptsis V. Tambur A. R. (2022). HLA-DQ mismatches lead to more unacceptable antigens, greater sensitization, and increased disparities in repeat transplant candidates. J. Am. Soc. Nephrol. 33 (12), 2293–2305. 10.1681/ASN.2022030296 36450598 PMC9731640

[B16] Johansen K. L. Chertow G. M. Foley R. N. Gilbertson D. T. Herzog C. A. Ishani A. (2021). US renal data system 2020 annual data report: epidemiology of kidney disease in the United States. Am. J. Kidney Dis. 77, A7–A8. 10.1053/j.ajkd.2021.01.002 33752804 PMC8148988

[B17] Kabani R. Quinn R. R. Palmer S. Lewin A. M. Yilmaz S. Tibbles L. A. (2014). Risk of death following kidney allograft failure: a systematic review and meta-analysis of cohort studies. Nephrol. Dial. Transplant. 29 (9), 1778–1786. 10.1093/ndt/gfu205 24895440

[B18] Kaplan B. Meier-Kriesche H. U. (2002). Death after graft loss: an important late study endpoint in kidney transplantation. Am. J. Transplant. 2 (10), 970–974. 10.1034/j.1600-6143.2002.21015.x 12482151

[B19] Knoll G. Campbell P. Chasse M. Fergusson D. Ramsay T. Karnabi P. (2022). Immunosuppressant medication use in patients with kidney allograft failure: a prospective multi-center Canadian cohort study. J. Am. Soc. Nephrol. 33 (6), 1182–1192. 10.1681/ASN.2021121642 35321940 PMC9161795

[B20] Knoll G. Muirhead N. Trpeski L. Zhu N. Badovinac K. (2005). Patient survival following renal transplant failure in Canada. Am. J. Transplant. 5 (7), 1719–1724. 10.1111/j.1600-6143.2005.00921.x 15943631

[B21] Kosmoliaptsis V. Mallon D. H. Chen Y. Bolton E. M. Bradley J. A. Taylor C. J. (2016). Alloantibody responses after renal transplant failure can Be better predicted by donor–recipient HLA amino acid sequence and physicochemical disparities than conventional HLA matching. Am. J. Transplant. 16 (7), 2139–2147. 10.1111/ajt.13707 26755448 PMC5021128

[B22] Kramer C. S. M. Koster J. Geert | Haasnoot W. Roelen D. L. Claas F. H. J. (2020). HLA-EMMA: a user-friendly tool to analyse HLA class I and class II compatibility on the amino acid level. Available from: https://www.phla3d.com.br/. 10.1111/tan.13883 PMC7317360 32227681

[B23] Lam N. N. Boyne D. J. Quinn R. R. Austin P. C. Hemmelgarn B. R. Campbell P. (2020). Mortality and morbidity in kidney transplant recipients with a failing graft: a matched cohort study. Can. J. Kidney Health Dis. 7, 2054358120908677. 10.1177/2054358120908677 32313663 PMC7158256

[B24] Lan J. H. (2024). Assessment of novel therapeutics to improve access to transplantation for highly sensitized patients in a shifting clinical landscape. J. Am. Soc. Nephrol. 35 (3), 259–260. 10.1681/ASN.0000000000000302 38303118 PMC10962892

[B25] Lee H. Min J. W. Kim J. I. Moon I. S. Park K. H. Yang C. W. (2016). Clinical significance of HLA-DQ antibodies in the development of chronic antibody-mediated rejection and allograft failure in kidney transplant recipients. Medicine 95 (11), e3094. 10.1097/MD.0000000000003094 26986147 PMC4839928

[B26] Lucisano G. Brookes P. Santos-Nunez E. Firmin N. Gunby N. Hassan S. (2019). Allosensitization after transplant failure: the role of graft nephrectomy and immunosuppression – a retrospective study. Transpl. Int. 32 (9), 949–959. 10.1111/tri.13442 30980556

[B27] Martin K. Cantwell L. Barraclough K. A. Lian M. Masterson R. Hughes P. D. (2021). Prolonged immunosuppression does not improve risk of sensitization or likelihood of retransplantation after kidney transplant graft failure. Transpl. Int. 34 (11), 2353–2362. 10.1111/tri.13998 34320262

[B28] Nimmo AMSA McIntyre S. Turner D. M. Henderson L. K. Battle R. K. (2018). The impact of withdrawal of maintenance immunosuppression and graft nephrectomy on HLA sensitization and calculated chance of future transplant. Transpl. Direct 4 (12), e409. 10.1097/TXD.0000000000000848 PMC6283087 30584590

[B29] Ojo A. O. Wolfe R. A. Agodoa L. Y. Held P. J. Port F. K. Leavey S. F. (1998). Prognosis after primary renal transplant failure and the beneficial effects of repeat transplantation: multivariate analyses from the United States Renal Data System. Transplantation 66 (12), 1651–1659. 10.1097/00007890-199812270-00014 9884254

[B30] Otten H. G. Calis J. J. A. Keşmir C. van Zuilen A. D. Spierings E. (2013). Predicted indirectly recognizable HLA epitopes presented by HLA-DR correlate with the *de novo* development of donor-specific HLA IgG antibodies after kidney transplantation. Hum. Immunol. 74 (3), 290–296. 10.1016/j.humimm.2012.12.004 23232063

[B31] Pham P. T. Everly M. Faravardeh A. Pham P. C. (2015). Management of patients with a failed kidney transplant: dialysis reinitiation, immunosuppression weaning, and transplantectomy. World J. Nephrol. 4 (2), 148–159. 10.5527/wjn.v4.i2.148 25949929 PMC4419125

[B32] Rao P. S. Schaubel D. E. Jia X. Li S. Port F. K. Saran R. (2007). Survival on dialysis post-kidney transplant failure: results from the scientific registry of transplant recipients. Am. J. Kidney Dis. 49 (2), 294–300. 10.1053/j.ajkd.2006.11.022 17261432

[B33] Rao P. S. Schaubel D. E. Wei G. Fenton S. S. A. (2006). Evaluating the survival benefit of kidney retransplantation. Transplantation 82 (5), 669–674. 10.1097/01.tp.0000235434.13327.11 16969291

[B34] Schold J. D. Augustine J. J. Huml A. M. O’Toole J. Sedor J. R. Poggio E. D. (2020). Modest rates and wide variation in timely access to repeat kidney transplantation in the United States. Am. J. Transplant. 20 (3), 769–778. 10.1111/ajt.15646 31599065 PMC7204603

[B35] Senev A. Coemans M. Lerut E. Van Sandt V. Kerkhofs J. Daniëls L. (2020). Eplet mismatch load and *de novo* occurrence of donor-specific anti-HLA antibodies, rejection, and graft failure after kidney transplantation: an observational cohort study. J. Am. Soc. Nephrol. 31 (9), 2193–2204. 10.1681/ASN.2020010019 32764139 PMC7461684

[B36] Vincenti F. Bestard O. Brar A. Cruzado J. M. Seron D. Gaber A. O. (2023). Isatuximab monotherapy for desensitization in highly sensitized patients awaiting kidney transplant. J. Am. Soc. Nephrol. 35, 347–360. 10.1681/asn.0000000000000287 38147137 PMC10914196

[B37] Wiebe C. Kosmoliaptsis V. Pochinco D. Gibson I. W. Ho J. Birk P. E. (2019). HLA-DR/DQ molecular mismatch: a prognostic biomarker for primary alloimmunity. Am. J. Transplant. 19 (6), 1708–1719. 10.1111/ajt.15177 30414349 PMC6563434

[B38] Wiebe C. Kosmoliaptsis V. Pochinco D. Taylor C. J. Nickerson P. (2018). A comparison of HLA molecular mismatch methods to determine HLA immunogenicity. Transplantation 102 (8), 1338–1343. 10.1097/TP.0000000000002117 29443827 PMC6072378

[B39] Wiebe C. Nevins T. E. Robiner W. N. Thomas W. Matas A. J. Nickerson P. W. (2015). The synergistic effect of class II HLA epitope-mismatch and nonadherence on acute rejection and graft survival. Am. J. Transplant. 15 (8), 2197–2202. 10.1111/ajt.13341 26095765

[B40] Wiebe C. Rush D. N. Nevins T. E. Birk P. E. Blydt-Hansen T. Gibson I. W. (2017). Class II eplet mismatch modulates tacrolimus trough levels required to prevent donor-specific antibody development. J. Am. Soc. Nephrol. 28 (11), 3353–3362. 10.1681/ASN.2017030287 28729289 PMC5661295

[B41] Willicombe M. Brookes P. Sergeant R. Santos-Nunez E. Steggar C. Galliford J. (2012). *De novo* DQ donor-specific antibodies are associated with a significant risk of antibody-mediated rejection and transplant glomerulopathy. Transplantation 94 (2), 172–177. 10.1097/TP.0b013e3182543950 22735711

