# Supplementary figures and images for: Clinical relevance of HLA-DQ eplet mismatch and maintenance immunosuppression with risk of allosensitization after kidney transplant failure
Source: Front Genet. 2024 Apr 4;15:1383220. doi: 10.3389/fgene.2024.1383220 (PMC11024336; doi:10.3389/fgene.2024.1383220)

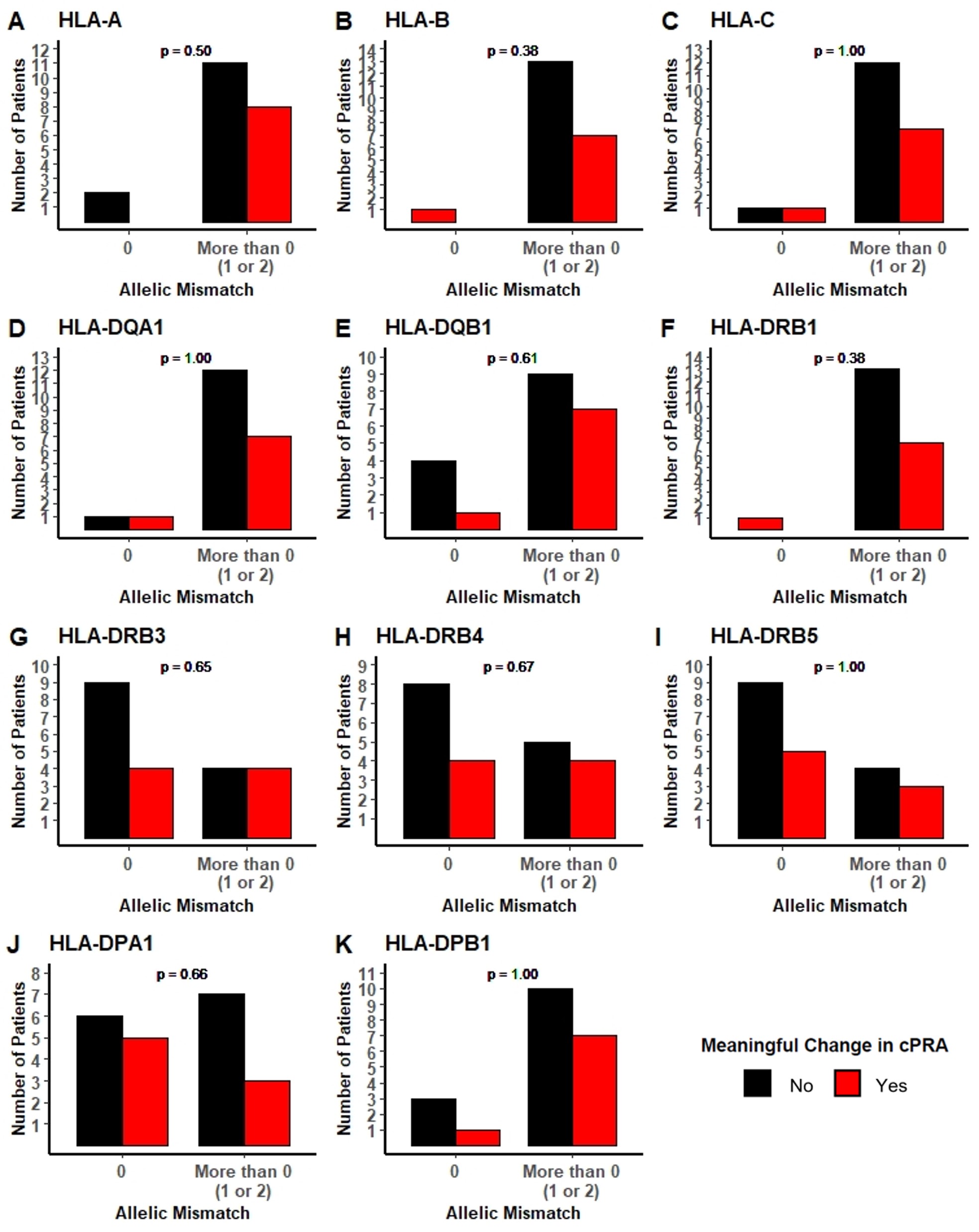

Supplement: Supplementary file 1 [file Image3.JPEG]

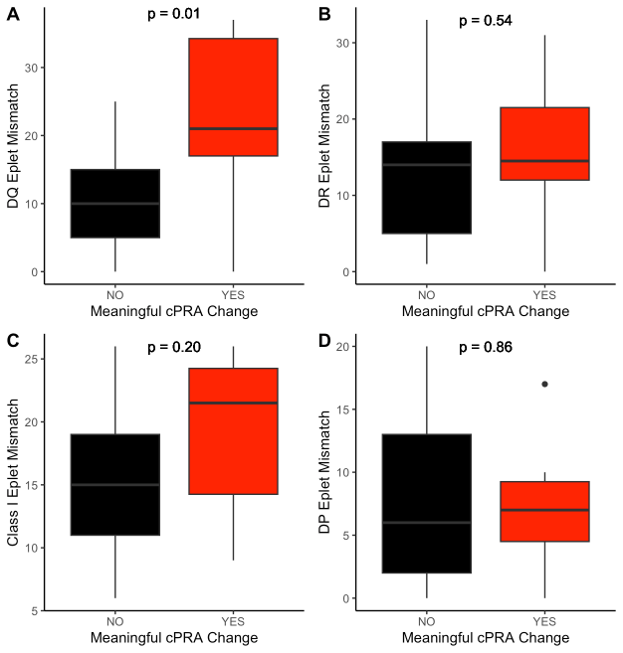

Supplement: Supplementary file 2 [file Image1.TIFF]

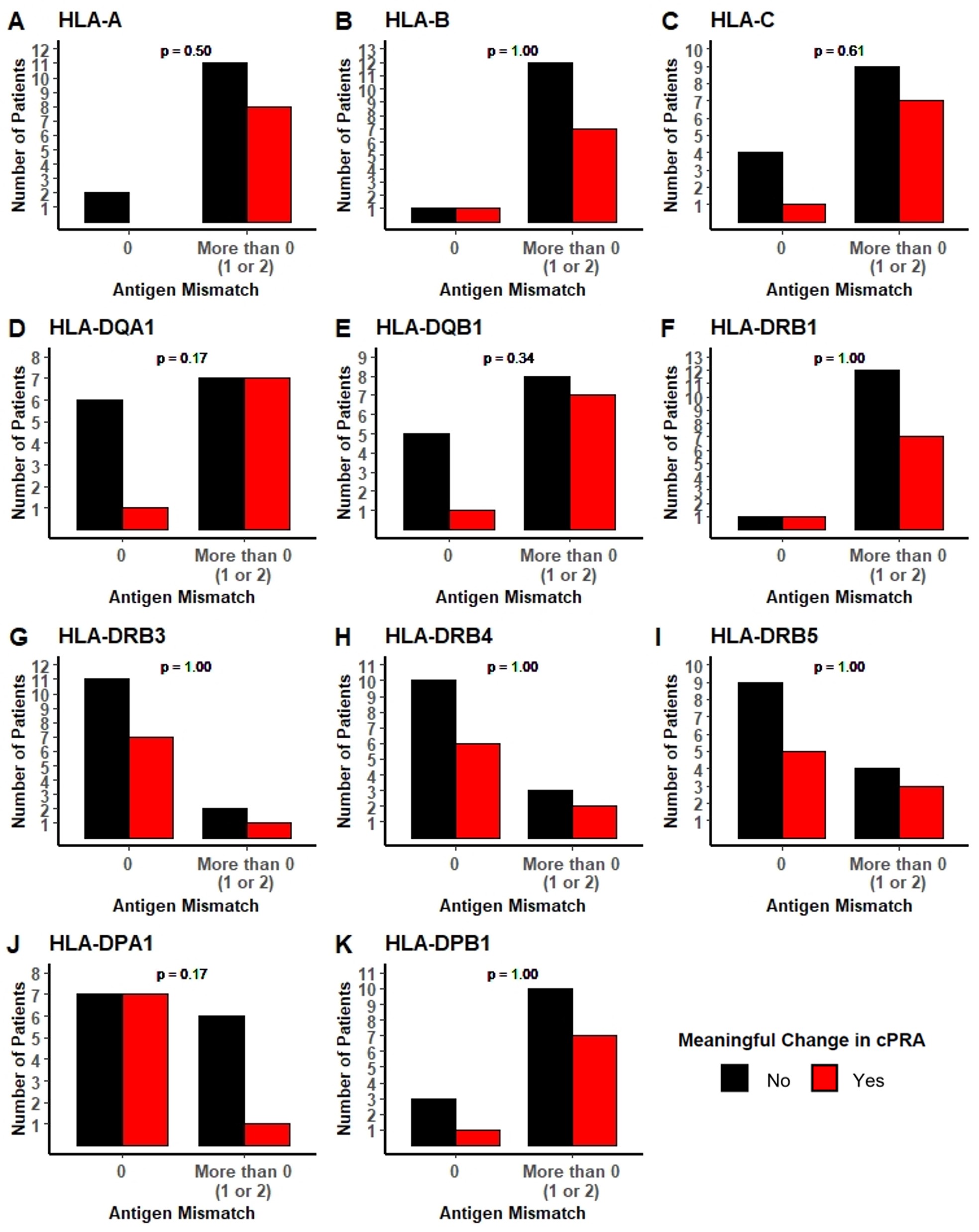

Supplement: Supplementary file 3 [file Image2.JPEG]

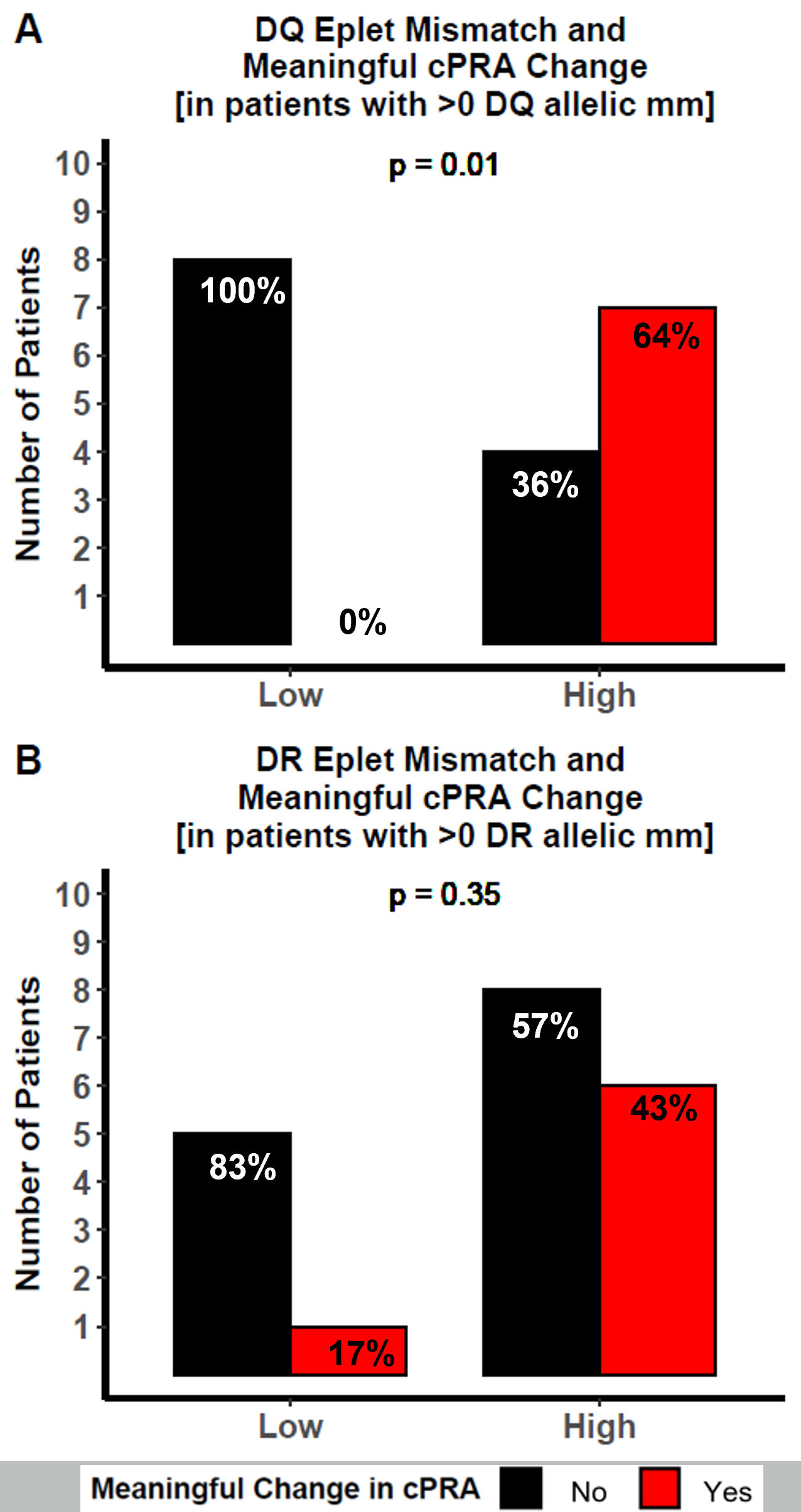

Supplement: Supplementary file 4 [file Image4.TIFF]
